# New developments in the *ATSAS* program package for small-angle scattering data analysis

**DOI:** 10.1107/S0021889812007662

**Published:** 2012-03-15

**Authors:** Maxim V. Petoukhov, Daniel Franke, Alexander V. Shkumatov, Giancarlo Tria, Alexey G. Kikhney, Michal Gajda, Christian Gorba, Haydyn D. T. Mertens, Petr V. Konarev, Dmitri I. Svergun

**Affiliations:** a European Molecular Biology Laboratory, Hamburg Unit, EMBL c/o DESY, Notkestrasse 85, Hamburg 22607, Germany

**Keywords:** isotropic scattering, small-angle scattering, data analysis, biological macromolecules, structural modelling, *ATSAS*, computer programs

## Abstract

New developments in the program package *ATSAS* (version 2.4) for the processing and analysis of isotropic small-angle X-ray and neutron scattering data are described. They include (i) multiplatform data manipulation and display tools, (ii) programs for automated data processing and calculation of overall parameters, (iii) improved usage of high- and low-resolution models from other structural methods, (iv) new algorithms to build three-dimensional models from weakly interacting oligomeric systems and complexes, and (v) enhanced tools to analyse data from mixtures and flexible systems. The new *ATSAS* release includes installers for current major platforms (Windows, Linux and Mac OSX) and provides improved indexed user documentation. The web-related developments, including a user discussion forum and a widened online access to run *ATSAS* programs, are also presented.

## Abstract 2

The paper presents new developments and amendments to the *ATSAS* package (version 2.4) for processing and analysis of isotropic small-angle scattering data.

## 1. Introduction   

Small-angle scattering (SAS) of X-rays (SAXS) and neutrons (SANS) is widely used to study the low-resolution structure of diverse noncrystalline systems in physics, materials science and biology (Feigin & Svergun, 1987 ▶). Modern SAS instrumentation, including high-flux dedicated synchrotron radiation and neutron beamlines and advanced laboratory X-ray sources, allows one to collect high-quality experimental data (Hura *et al.*, 2009 ▶; Round *et al.*, 2008 ▶; Teixeira *et al.*, 2008 ▶; Toft *et al.*, 2008 ▶). The hardware developments are accompanied by substantial progress in methods for data interpretation, which are especially noticeable in the analysis of isotropic scattering. One of the most rapidly developing areas of research using SAS is the structural characterization of macromolecular solutions, facilitated by recent advances in analysis approaches. In structural biology, low-resolution shapes of macromolecules are now routinely reconstructed *ab initio*, and automated procedures to construct rigid-body models of complex particles are well established (Mertens & Svergun, 2010 ▶). The analysis methods developed for biological macromolecules have also been applied to modern nanomaterials research (Bronstein *et al.*, 2010 ▶; Shtykova *et al.*, 2010 ▶), further widening the range of advanced applications of the technique.

There are several programs available that focus on different aspects of SAS data processing and analysis from isotropic systems. The *IRENA* package (Ilavsky & Jemian, 2009 ▶) is based on the proprietary *IGOR Pro* (http://www.wavemetrics.com) computing environment and is primarily oriented towards data analysis from nonbiological systems; however, certain tools such as Guinier and Porod fits and the pair-distance distribution functions are also applicable for monodisperse solution scattering. The *SASfit* package (http://kur.web.psi.ch/sans1/SANSSoft/sasfit.html) provides means of analysing and plotting SAS data, calculating the overall structural parameters, and fitting structural models to data from contrast variation experiments. *BioXTAS RAW* (Nielsen *et al.*, 2009 ▶) is designed for automated and manual reduction and primary analysis of isotropic SAXS data and allows one to calculate the pair-distance distribution functions.

Among the publicly available packages, *ATSAS* (Konarev *et al.*, 2006 ▶; Petoukhov *et al.*, 2007 ▶) is perhaps the most comprehensive collection of tools covering the major data manipulation and interpretation tasks. Since 2003, *ATSAS* has been downloaded more than 30 000 times by about 4500 users from over 1500 laboratories. As of August 2007, the major *ATSAS* programs also became accessible online, and this service is now actively used by the community. During 2007–2011, *ATSAS* programs were cited in about 1100 publications, with over 700 devoted to biological SAS, constituting more than a half of all publications on biological solution SAS worldwide.

**Figure 1:**
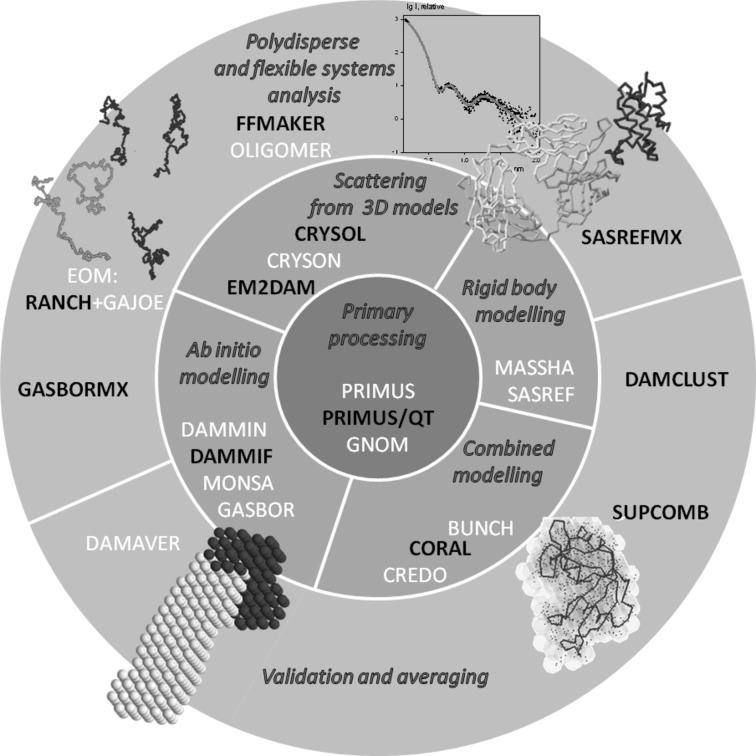
Major analysis tasks and computational modules of the *ATSAS* program package. Newly developed programs and the ones significantly enhanced in the 2.4 release are given in black capital letters.

The *ATSAS* programs are being constantly developed to widen the functionality and to improve the performance, speed and reliability of the suite. The overall structure of the current *ATSAS* version (2.4) is presented in Fig. 1 ▶, together with brief descriptions of the functions of the individual groups of programs. The present paper describes the major recent developments in *ATSAS*, including improvements in the existing programs as well as addition of new programs. The main aim of the paper is to provide a concise description of the new *ATSAS* features, and we shall therefore give only a brief account of the functionality of the new or improved modules. For detailed descriptions of the existing programs belonging to *ATSAS*, users are referred to the original papers (see Petoukhov *et al.*, 2007 ▶, and references therein). The novel computational methods employed in the new *ATSAS* programs together with test cases validating these approaches are presented in detail elsewhere (M. V. Petoukhov, I. M. L. Billas, D. Moras & D. I. Svergun, in preparation). In the present paper we shall also describe the new and improved *ATSAS*-related services, including multiplatform installers, documentation in the form of an online help, and a web user forum for discussing and resolving the problems occurring when running the software.

## 2. Primary data processing   

### 2.1. Cross-platform applications with graphical user interface for data manipulation   

**Figure 2:**
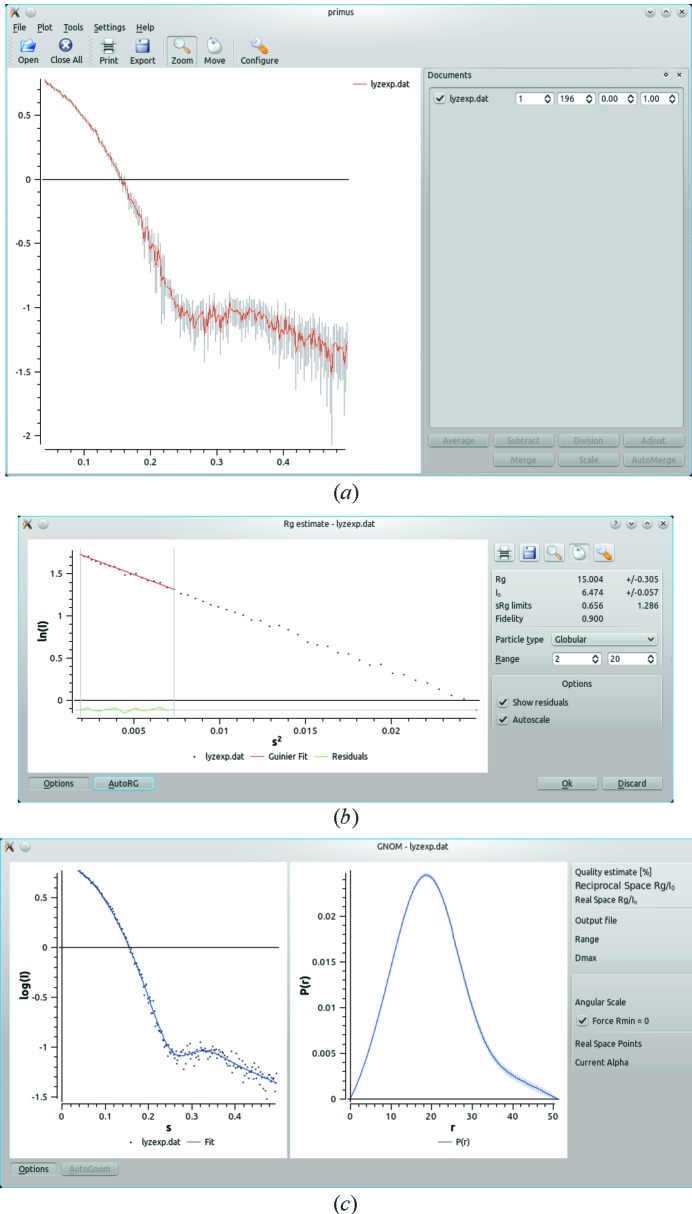
Screenshots of the cross-platform *PRIMUS*. (*a*) One experimental profile with errors is displayed. The ‘Documents’ panel may be undocked and moved freely. It may hold a (virtually) unlimited number of files, provides access to the most frequently used operations and keeps a list of recently used files. (*b*) Interactive estimation of the radius of gyration. Advanced options are hidden by default; the display of residuals may be enabled or disabled. Zooming, panning and export to various graphics formats are available. (*c*) Estimation of the distance distribution; the fit to the data and the *P*(*r*) function are shown next to each other and instantly updated if any of the configuration options is changed. Advanced options are hidden by default.

The first release of the graphical data analysis program *PRIMUS* (Konarev *et al.*, 2003 ▶) was designed specifically for the Microsoft Windows operating system. From that time there has been a steady increase in demand for versions of *PRIMUS* that also run natively on alternative platforms (*i.e.* Linux, Mac). With the release of *ATSAS 2.4*, the first test version of a cross-platform implementation of *PRIMUS* is provided, which also includes a cross-platform version of the data visualization utility *SASPLOT*. The applications are based on the graphical user interface toolkit Qt (http://www.iucr.org/resources/commissions/crystallographic-computing/newsletters/1/cross-platform-gui-development). Though the Qt *PRIMUS* implementation has at present fewer features than its Windows counterpart, basic data visualization in a variety of scaling schemes {*e.g.* absolute scale, log, log–log, Kratky [*I*(*s*)*s* ^2^ *versus s*] and Porod [*I*(*s*)*s* ^4^ *versus s*]} is provided. Here, *I*(*s*) is the scattering intensity and *s* is the scattering vector magnitude, defined as *s* = 4πsinθ/λ, where λ is the wavelength and 2θ is the scattering angle. Advanced zooming and panning of the canvas is available within the data manipulation screen of Qt *PRIMUS*. As in the original implementation, basic operations for file manipulation and processing are present, including averaging, subtraction, adjustment, scaling and merging of data sets. The new *PRIMUS* can load a (virtually) unlimited number of files, compared to the previous limitation of 12, and keeps a list of recently used files for quick access. Fig. 2 ▶(*a*) shows the *Qt PRIMUS* data analysis window on Linux with one loaded data file. The ‘Tools’ menu currently allows (i) estimation of the radius of gyration (Fig. 2 ▶ *b*), where the *AUTORG* utility (Petoukhov *et al.*, 2007 ▶) is used to obtain an initial estimate automatically, and (ii) computation of the distance distribution function *P*(*r*), *i.e.* a front end for the indirect Fourier transformation program *GNOM* (Svergun, 1992 ▶). Interactive monitoring of *GNOM* results is provided where both the computed *P*(*r*) function and the fit to the data are displayed side by side (Fig. 2 ▶ *c*). Both plots are updated in real time as the user adjusts the fitting parameters (*e.g. D*_max_, the number of data points, and the regularization parameter, α) and the quality of the solution using perceptual criteria is displayed. This visualization makes the use of *GNOM* much more intuitive and in some cases it helps to refine the results obtained by automated processing. Additional tools are presently being incorporated to both reach and improve upon the full functionality of the original Windows-based application. The new *SASPLOT* provides the basic plotting capabilities of the *ATSAS* file formats (*e.g.* *.dat, *.int, *.fit and *.fir), and, like *PRIMUS*, supports multiple scaling schemes and zooming/panning. An improved configuration of the plot canvas and export capabilities to various image formats are provided.

### 2.2. Automated merging and extrapolation to infinite dilution   

In most structural studies of macromolecular solutions using SAS it is indispensible to remove the scattering contribution due to interparticle interactions and to extrapolate the data to infinite dilution. Manual procedures for the merging and the extrapolation of multiple SAXS data sets are available (*e.g.* using the ‘Zerconc’ option in *PRIMUS*), but such an interactive step of course can not be incorporated into automated pipelines. A new program, *AUTOMERGE*, has been designed for automatic extrapolation and merging of several data sets collected on the same sample at different solute concentrations. *AUTOMERGE* scales a set of input scattering profiles to obtain a reliable profile overlap and checks that the sample identity is qualitatively consistent across the series (*i.e.* the scattering patterns at higher angles coincide well). It further determines the intensity extrapolated to infinite dilution 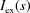 assuming that there exists a linear relationship between the intensity and solute concentration, the ‘concentration effect’, represented as 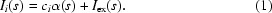
Here 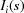 is the scattering intensity at a concentration 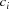 and 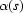 is a term describing the concentration effect (which is defined by intermolecular interactions and does not depend on the concentration at the considered 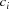 range). *AUTOMERGE* starts by finding the optimal fitting range where most scattering profiles match after appropriate scaling, and the scaled files are used for a pointwise extrapolation to zero concentration (Feigin & Svergun, 1987 ▶). The validity of the extrapolation to zero concentration is controlled by diagnostic tools that compare expected output with the actual estimate. The primary quality check of the extrapolation is performed by comparison of the radius of gyration (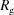) value for the extrapolated curve and the linear extrapolation of 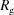 values from all input profiles. Additionally, a check on the pointwise error of linear extrapolation is performed, using standard procedures for least-squares approximation. The information gain is also estimated from the difference between an extrapolated point and any of the input points across a region of the extrapolated curve, where the minimum difference is larger than that expected for a numerical error of extrapolation. The extrapolated curve is merged with the pointwise averaged curve by choosing a merging point within the optimal fitting range. More details of the *AUTOMERGE* algorithm will be given elsewhere (M. Gajda & D. I. Svergun, in preparation).

### 2.3. Automated determination of the particle volume and molecular mass   

The molecular mass (MM) of the particle is one of the major overall parameters that can be directly deduced from the experimental solution scattering data. This parameter allows one to determine the oligomeric state of homomultimers at given conditions or reach conclusions about the possible complex formation for a mixture of several distinct components. The MM is obtained from the forward scattering of the sample 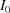 (by comparison with a reference, *e.g.* of bovine serum albumin), which is normalized by the sample concentration *c*, so that the accuracy of the MM is limited by the reliability of the measured concentration *c*. In some cases obtaining an estimate of *c* is difficult (*e.g.* for proteins containing few aromatic residues), and the typical accuracy of the MM determination in practice is about 10–15% (Mylonas & Svergun, 2007 ▶). Alternatively, the MM may be estimated from the scattering data based on the excluded (*i.e.* hydrated) particle volume. The latter is computed without normalization of the intensity (Porod, 1982 ▶) from the experimental data, 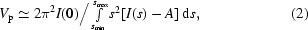
and the MM can be assessed from 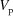 provided the partial specific volume and hydration of the particle are known. To compute the 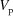 value, an appropriate constant [*A* in equation (2)] has to be subtracted from each data point to force the 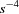 decay of the intensity at higher angles following Porod’s (1982 ▶) law for homogeneous particles. This procedure yields a ‘shape scattering’ curve and corrects for the unwanted scattering contribution from the internal structure.

Direct use of equation (2) to obtain 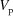 is difficult as the results may strongly depend on the experimental data range (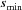, 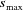), and moreover, the estimation of *A* is a nontrivial procedure as a result of noise. However, an enforced 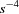 decay of the intensity at higher angles allows one to extrapolate the scattering curve to infinity and therefore to diminish the inaccuracy of the integral calculation in equation (2). An empirical correction factor for the calculation of the Porod integral given the limited integration range was devised by Rolbin *et al.* (1973 ▶).

The exact relationship between MM and 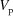 varies for different proteins depending on a combination of several factors, *e.g.* particle anisometry, flexibility *etc*. To design an algorithm for a reliable automated calculation of 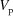, a processed scattering intensity [*e.g.* that produced by the program *GNOM* (Svergun, 1992 ▶)] is used instead of the raw experimental data. The major aim of the present algorithm was not to provide the most accurate evaluation of 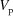 but instead to devise the best strategy for an automated calculation of the 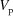 value yielding the most stable ratio to MM in terms of the lowest standard deviation of the calculated MM compared to the expected value. For this purpose, atomic models of 53 proteins ranging from 14 to 500 kDa were taken from the Protein Data Bank (PDB; Bernstein *et al.*, 1977 ▶) (of these, 34 and 11 proteins were solved by X-ray and electron crystallography, respectively, and eight were obtained by NMR), and their theoretical scattering intensities were computed using the program *CRYSOL* (Svergun *et al.*, 1995 ▶). It was found (see supplementary materials^1^) that the scattering data range up to about 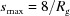 is optimal for a reliable computation of 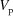. This upper limit in most cases approximately corresponds to the second minimum in the Porod plot [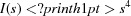 *versus* 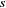]. This range is also suitable for the automated determination of the particle maximum size and the *P*(*r*) function as well as evaluation of the processed back-transformed intensity using the program *AUTOGNOM* (Petoukhov *et al.*, 2007 ▶). Using this interval, the average ratio between MM and 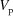 is 0.625, yielding an accuracy of the MM estimate nearing 20%, without clear systematic correlation between the ratio, the size of the protein and its anisometry (see supplementary materials). Based on this approach the program *AUTOPOROD* was developed, which (i) automatically runs the *AUTORG* (Petoukhov *et al.*, 2007 ▶) and *AUTOGNOM* (Petoukhov *et al.*, 2007 ▶) tools to find the first good point in the data 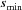 (*i.e.* the closest-to-the-origin angular interval displaying a valid Guinier behaviour) and the 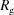 value according to Guinier’s law and to generate the regularized profile, (ii) determines and subtracts the appropriate background constant *A* to enforce the Porod behaviour, and (iii) evaluates 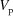 using the data range from 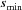 to 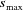 and computes the MM employing the above parameters.

Recently, a web portal ‘SAXS *MoW*’ was introduced (Fischer *et al.*, 2010 ▶), which employs the Porod volume to obtain an assessment of MM. The portal features a convenient user interface, where the scattering data pre-processed by *GNOM* can be uploaded. The angular range over which to estimate the volume is selected interactively and the MM is calculated using an empirical ratio. In its present form, no constant subtraction is performed, which could make the results sensitive to possible deviations at higher angles (*e.g.* as a result of variations in the background scattering). Overall, *MoW* appears to be a useful interactive tool, whereas *AUTOPOROD* is designed for both interactive use and as a component of automated pipelines.

The automated tools mentioned above enable data processing without human intervention and can be employed in pipelines for the primary data analysis. Additional modularized tools for data manipulation are also distributed as a part of the *ATSAS* package. These tools include (i) *DATOP*, a utility to perform operations on data files in various formats, *e.g.* addition or subtraction of two files, multiplication by a constant *etc*., (ii) *DATCMP*, an application to compare two data files in various formats, and (iii) *DATAVER*, a tool to average two or more data files, again in various file formats. Thanks to their modularity, the output of one application may be directly transferred into the input of another application, allowing for an easy construction of customized workflows. Fully automated pipelines employing all the presented modules are currently running at the SAXS beamlines of EMBL in Hamburg and also at the ID14-3 beamline at ESRF in Grenoble. The modularized tools are based on the libsaxsdocument library, which was made available under the GNU public license LGPLv3, and the source codes can be obtained from http://saxsview.sf.net. Support for new file formats can easily be added, making the pipelines customizable for other instruments.

## 3. The use of structural models from complementary methods   

In this section, improvements in the programs to use the models provided by complementary structural methods, including X-ray crystallography (MX), nuclear magnetic resonance (NMR) and electron microscopy (EM), are presented.

### 3.1. X-ray and neutron scattering calculation from high-resolution structures   

The programs *CRYSOL* (Svergun *et al.*, 1995 ▶) for X-rays and *CRYSON* (Svergun *et al.*, 1998 ▶) for neutrons evaluate the solution scattering from macromolecules with known atomic structure and fit a predicted curve 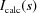 to experimental scattering data 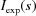 by minimizing the discrepancy (Feigin & Svergun, 1987 ▶)
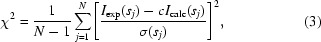
where *c* is a scaling factor, *N* is the number of points and σ denotes the experimental errors. In the fitting process, the excess scattering density of the hydration shell, the average atomic group radius and the related total excluded volume can be adjusted. With the recent progress in high-resolution structure determination and advances in structure prediction and docking algorithms, tremendous numbers of structural models are becoming available. The screening of multiple models against experimental scattering data (typically SAXS) is often performed to select the best configuration in solution. The performance of these programs is crucial when applied to large numbers of structures and to deal with the increased number of angular data points in scattering profiles resulting from the improved resolution of detectors employed at the modern SAXS beamlines (*e.g. PILATUS* from *DECTRIS*; http://pilatus.web.psi.ch/pilatus.htm). In order to speed up *CRYSOL* calculations, experimental scattering intensities and associated errors are automatically remapped into a sparser grid for the search of the best fitting parameters. Depending on the number of experimental points, the regridding operation speeds up the fitting procedure by up to a factor of five. The final fits are recalculated for the optimum parameters for the original experimental data points.

Practice shows that in some cases (*e.g.* as a result of buffer mismatch) the higher-angle positions of the scattering data may contain systematic deviations, which can be accounted for by subtraction/addition of a constant term to the experimental data. An option of background constant adjustment has been added to *CRYSOL* to allow for the correction of such over- or under-subtracted buffer signal. A linear least-squares minimization with boundaries (Lawson & Hanson, 1995 ▶) is used to find the scaling coefficient and the background constant value when fitting a theoretical curve to experimental data.

Typically, *CRYSOL* and *CRYSON* skip all H atoms present in the PDB files and instead make an assignment of the number of bound H atoms for each atomic group based on the chemical compound library (ftp://ftp.wwpdb.org/pub/pdb/data/monomers/components.cif) in order to compute the scattering. If a full-atom model containing all H (or deuterium) atoms is available, the user has the option to take all the atoms ‘as is’, which can be specified in both interactive and batch modes [in the latter case the input parameters are specified on the command line (Konarev *et al.*, 2006 ▶)]. Since the remediation of the PDB archive in 2007–2008 (http://www.rcsb.org/pdb/static.do?p=general_information/news_publications/index.html) the nomenclature of many heteroatoms had been changed, and the assignments of bound hydrogen to an atom became ambiguous, such that the hydrogen assignment may be incorrect in some cases. To resolve this problem, both the new (after version 3.1) and the old (before version 3.0) PDB formats are now supported. By default, the new format is assumed, but the user can also enforce the old format by using the ‘/old’ key in the command line input.

### 3.2. The use of EM maps   

SAXS data are often used for validation and comparison with electron microscopy (EM) reconstructions (Andersen *et al.*, 2006 ▶; Tidow *et al.*, 2007 ▶; Vestergaard *et al.*, 2005 ▶). To conveniently work with EM models we developed a program *EM2DAM* (electron microscopy density map to dummy atom model), which converts an EM density map into a bead model in a PDB-like format (Bernstein *et al.*, 1977 ▶). The latter model can be used for the calculation of the theoretical intensity and fitting to experimental scattering curves, *e.g.* using *CRYSOL*. If the EM map file follows the MRC format (Crowther *et al.*, 1996 ▶), the user should only supply the threshold value defining the particle in the EM map, while all other parameters (number of voxels, voxel size *etc.*) are extracted from the header of the MRC file. It is also possible to read other formats [*CCP4* (Collaborative Computational Project, No. 4, 1994 ▶), *SPIDER* http://www.wadsworth.org/spider_doc/spider/docs/spider.html *etc.*], by specifying these parameters in the interactive mode. The EM-based dummy atom model can also be mildly refined. If the ‘--damform’ option is selected, the resulting model can be used as an initial search volume in *DAMMIN* (Svergun, 1999 ▶), whereby the surface beads (within a user-specified cutoff) can change their phase from particle to solvent during *DAMMIN* refinement while the core beads remain fixed.

## 4. Evaluation of multiple SAS-based reconstructions   

**Figure 3:**
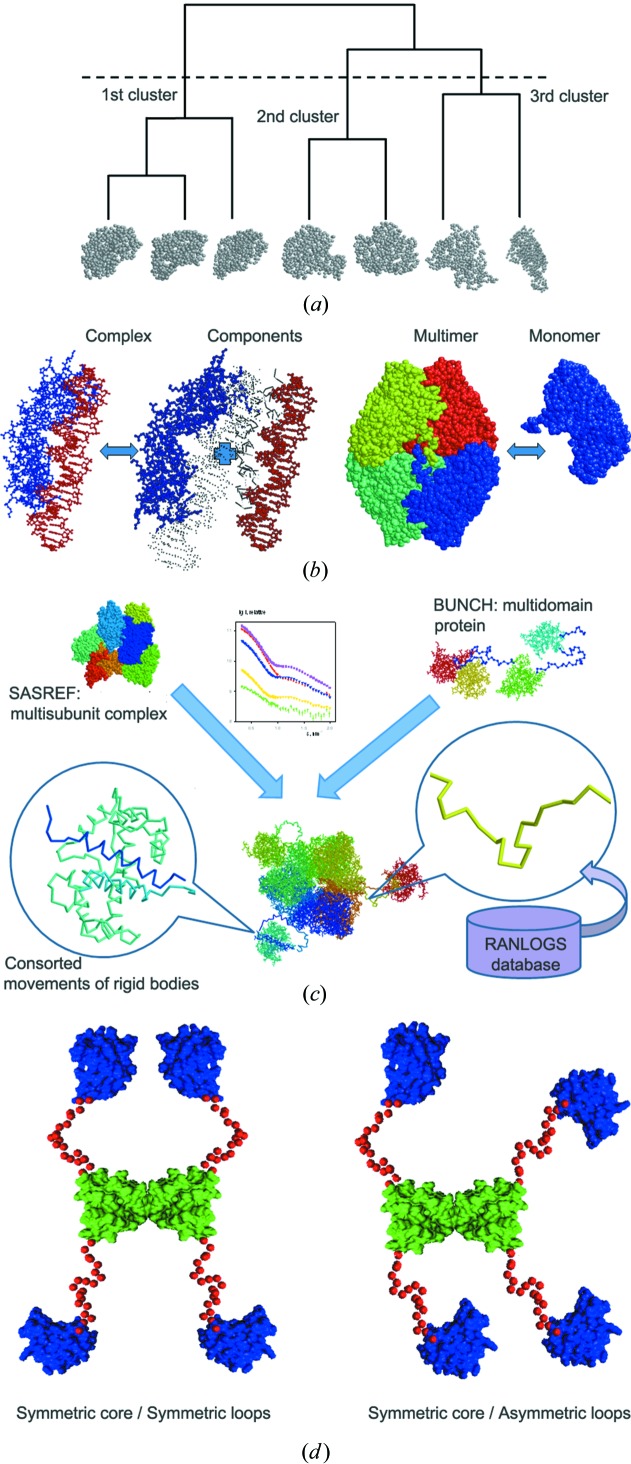
Schematic representation of some new algorithms introduced in *ATSAS 2.4*. (*a*) Clustering of multiple models by *DAMCLUST*. The program suggests the optimal way of splitting multiple models into groups based on the distance criteria (either NSD or r.m.s.d.). In the presented example, seven models are split into three groups (indicated by the dashed line), whereby there are three models in the first group while the second and the third groups contain two models each. (*b*) Modelling algorithms for transient complexes (*SASREFMX*) and unstable oligomers (*GASBORMX*/*SASREFMX*) provide means of three-dimensional structure analysis of equilibrium mixtures, if no monodisperse samples can be obtained. (*c*) Rigid-body modelling of complexes with addition of missing loops by *CORAL*, which combines the capabilities of *SASREF* and *BUNCH*. The modelling of complicated cases with incomplete structures of the subunits is possible, whereby the *RANLOGS* library is used for selection of templates for missing linkers. (*d*) A new implementation of *RANCH* from the EOM package enables generation of random pools containing multimeric structures having fixed symmetric cores with or without imposed symmetry for the rest of the molecule.

Reconstruction of the three-dimensional structure from the one-dimensional scattering curve is generally ambiguous even at low resolution. Multiple runs of Monte Carlo-based minimization programs are needed to assess the uniqueness. One of the first attempts to analyse the convergence of multiple solutions was implemented in the program *DAMAVER* (Volkov & Svergun, 2003 ▶) for the post-processing of *ab initio* bead models. Based on a normalized spatial discrepancy (NSD; Kozin & Svergun, 2001 ▶) between individual models, the most typical model (having the lowest average NSD with respect to all the others) is selected. The outliers (with a significantly higher average NSD) are also identified and discarded. The remaining models are superimposed with the most typical one and averaged. The excluded outliers (which also fit the scattering data and fulfil other modelling restraints) may, however, provide alternative solutions, helping one to better evaluate the non-uniqueness, or, even worse, these ‘outliers’ may be the true positives. In a more versatile approach implemented in the program *DAMCLUST*, none of the models are discarded and instead all are clustered into groups, such that each group contains similar models (Fig. 3 ▶ *a*). The NSD is still used as a measure of dissimilarity between *ab initio* low-resolution structures; however, for clustering of rigid-body models with one-to-one correspondence between the atoms a root-mean-square deviation (r.m.s.d.) could also be applied. The evaluation of the effective distance (dissimilarity) between two clusters or a single model and a cluster as well as the optimal choice of the number of clusters is performed according to the algorithm proposed by Kelley *et al.* (1996 ▶). For each cluster, the most typical model is then selected and the averaged shape is built. Comparison between the representatives of individual clusters provides an idea of the possible ambiguity of the reconstruction. *DAMCLUST* can be used to analyse the *ab initio* models as an alternative to *DAMAVER* (especially useful for symmetric reconstructions, where the diversity of the models may be high), but also as a tool to clusterize the rigid-body models obtained by programs like *SASREF* or *BUNCH* (Petoukhov & Svergun, 2005 ▶).

The program *SUPCOMB* (Kozin & Svergun, 2001 ▶) for the alignment of two arbitrary low- or high-resolution models by minimizing the NSD dissimilarity measure has been updated, allowing one to take symmetry into account. Particularly, in the case of *Pn* symmetry and given the same direction of the *n*-fold symmetry axis for both models, the adjustments include only rotations and translations along this axis to preserve the common symmetry. For *Pn*2, superposition is done simply by the alignment of the corresponding symmetry axes of the two models. This feature enables one to keep the original symmetry in the averaged models generated by *DAMAVER* and *DAMCLUST*. The use of symmetry also improves the performance as fewer parameters are to be optimized during the alignment.

## 5. Analysis of equilibrium mixtures   

### 5.1. Mixtures with known scattering profiles of individual components   

For polydisperse systems without interparticle interactions, the scattering profile is a linear combination of the scattering intensities of individual components, weighted by their volume fractions 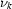 (Koch *et al.*, 2003 ▶): 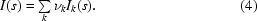
If the scattering patterns of all the mixture components are known, the volume fractions can be directly computed from SAS data. The program *OLIGOMER* (Konarev *et al.*, 2006 ▶) implements a non-negative linear least-squares algorithm (Lawson & Hanson, 1974 ▶) to find the volume fractions minimizing the discrepancy 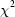 [equation (3)] between the predicted composite curve and the experimental data.

*OLIGOMER* requires an input file containing the intensities from individual components (‘form factors’). Manual preparation of such a file may be a cumbersome procedure, especially if data from heterogeneous models are to be put together. The program *FFMAKER* was developed to facilitate the creation of such a form-factor file. It is possible to combine the scattering intensities coming from different sources: (*a*) the theoretical scattering intensities from high-resolution PDB structures calculated by *CRYSOL* (Svergun *et al.*, 1995 ▶), (*b*) the experimental scattering curves and (*c*) the regularized (and desmeared if necessary) scattering curves obtained by *GNOM* (Svergun, 1992 ▶). *FFMAKER* can be run in interactive or batch mode, and for the latter the most important parameters can be supplied as arguments on the command line. *FFMAKER* provides also an option to evaluate multiple scattering intensities from an NMR ensemble stored in a single PDB file.

### 5.2. Accounting for polydispersity in three-dimensional modelling algorithms   

The requirement of monodispersity is a crucial prerequisite for a reliable three-dimensional model reconstruction from solution scattering data. In many cases monodispersity can be achieved by suitable preparation and handling of the sample or, for example, by using online high-performance liquid chromatography purification (Jensen *et al.*, 2010 ▶). Some samples, however, remain polydisperse despite all efforts, which makes a structural interpretation very difficult, even for well behaved systems with specific interactions. Typical examples are dynamic equilibria between monomers and higher oligomers in the case of single species or between bound and free components for low-affinity transient complexes. With rare exceptions (Blobel *et al.*, 2009 ▶; Vestergaard *et al.*, 2007 ▶), the options for the analysis of mixtures have been rather limited. Usually, it is only possible to predict the volume fractions of individual components if the models or scattering curves of all pure components are available (*e.g.* with *OLIGOMER*; see previous section). In some cases, one could try to perform three-dimensional modelling of the dominant component while neglecting the presence of the minor species (which however leads to systematic errors and possible misinterpretations). A novel option to account for dynamic equilibria was included in the *ATSAS* programs for both *ab initio* and rigid-body modelling (Fig. 3 ▶ *b*).

The *ab initio* program *GASBOR* represents the protein structure using a fixed number of dummy residues (DRs) and uses a simulated annealing (SA) protocol to obtain a spatial distribution of DRs that fits the experimental data (Svergun *et al.*, 2001 ▶). The original version of *GASBOR* reconstructs the shape assuming that the data correspond to a monodisperse system. The program was modified so that while fitting the data from the presumed homomultimer some fraction of a monomer is allowed. Assuming a symmetric quaternary structure of the multimer (where the symmetry group is defined by the oligomeric state, *e.g.* *P*2 for a dimer, *P*3 for a trimer *etc.*), the structure of the monomer is defined just by the asymmetric part of the entire DR ensemble. The interconnectivity is then required not only for the multimeric DR model but also for the DR portion forming the monomer. In the DR condensation procedure, the scattering intensities of the multimer and the monomer are computed from appropriate sets of DRs and the resulting profile from the mixture is computed as their linear combination. The volume fractions of the two components are defined by the least-squares fitting of the experimental curve using equation (4). Additionally, there is an option to provide the volume fractions (if they are known from other considerations), such that they will be fixed in the modelling. The feasible range of the volume fraction of the monomer for the reliable shape reconstruction is from about 0.2 to about 0.8 (independently of whether the fractions are fixed or not). Here, the lower limit is a result of the small contribution of the monomer intensity at lower volume fraction and the upper limit is explained by the need to estimate the maximum size of the oligomer, which is rather problematic at higher content of the monomers. The accuracy of the estimated volume fractions in our tests was typically about 15%.

The rigid-body modelling program *SASREF* (Petoukhov & Svergun, 2005 ▶) employs SA to position atomic models of individual subunits with respect to each other by moving and rotating them, so that an interconnected assembly without steric clashes is formed, while minimizing the discrepancy between the experimental SAS profile of the complex and the computed curve. The ability to account for possible polydispersity was added to *SASREF* in a yet more general way than that for *GASBOR*. The modelling was not limited to a multimer–monomer mixture, but instead arbitrary subsets of subunits can be selected as additional components (dissociation products). During the SA-driven modification of the mutual subunit arrangement, the experimental scattering curve is decomposed into the intensities computed from the entire multisubunit complex and from the specified subcomplex (*e.g.* a scenario is possible when a ternary complex partially dissociates into a binary part and a free third component). The volume fractions of the bound and the dissociated states in the mixture are determined by linear least-squares fitting, similar to the above *ab initio* case. The approach is useful even for very low affinity complexes with the volume fraction of the dissociated species within 0.90–0.15.

The two generalized programs called *GASBORMX* and *SASREFMX* are available in the *ATSAS 2.4* release. More detailed descriptions of their algorithms along with the test examples, assessment of the performance and limitations are presented elsewhere (M. V. Petoukhov, I. M. L. Billas, D. Moras & D. I. Svergun, in preparation).

## 6. Modelling of flexible systems   

### 6.1. A library of random loops   

High-resolution atomic models of proteins obtained by MX or NMR often lack some portions of the polypeptide chains. Typical examples are multidomain proteins with linkers, which are often split into individual domains to facilitate crystallization, or disordered regions (*e.g.* termini) not seen at high resolution owing to flexibility. Still, the regions missing in the high-resolution models do contribute to the scattering of the macromolecule in solution and their approximate configurations can be reconstructed (Petoukhov *et al.*, 2002 ▶; Petoukhov & Svergun, 2005 ▶). During such modelling, precise conformations of the missing terminal loop or interdomain linker are not required but approximate ‘placeholders’ should be added to the model to adequately compute the scattering intensity from the entire molecule. The use of pre-defined placeholders to connect individual domains would allow us to significantly speed up the calculations of rigid-body modelling algorithms. However, creation of native-like linkers with the given properties (*i.e.* length in terms of number of amino acids and end-to-end distance) is a time-consuming task.

A program *RANLOGS* (random loop generator and sorter), for generation and sorting of backbone-like loops of up to 100 amino acids in size, has been developed. As the loops generated are placeholders for either structured or less unstructured portions of the protein, the loops were generated *de novo*, without relying on any existing structures in the PDB. *RANLOGS* employs a simplified Cα-only representation of the loop, which is sufficient for the scattering intensity computation utilizing a dummy residues approach (Svergun *et al.*, 2001 ▶). The backbone is generated by a consecutive addition of Cα atoms at random positions with a 3.8 Å distance to the preceding one, while fulfilling two requirements: absence of steric clashes (distance to non-neighbouring Cα atoms must be more than 4 Å) and compliance of bond and dihedral angles with allowed angle combinations in a quasi-Ramachandran plot (Kleywegt, 1997 ▶). *RANLOGS* creates a pool of linkers of the sequence lengths within the specified range binned into the possible end-to-end distances (with the default discretization of 2 Å). For each combination of sequence length and end-to-end distance, several (typically 20) distinct random loops are selected. Such a sorted pool is convenient for the insertion of random loops between two anchor points with a pre-defined distance.

### 6.2. Rigid-body modelling combined with addition of missing fragments   

Several approaches have been developed in recent years for the modelling of multisubunit complexes and multidomain proteins against solution scattering data (Bertini *et al.*, 2010 ▶; Petoukhov & Svergun, 2005 ▶; Pons *et al.*, 2010 ▶). The efficiency of these approaches has been demonstrated in a number of applications to complicated biological systems (Boczkowska *et al.*, 2008 ▶; Gherardi *et al.*, 2006 ▶; Niemann *et al.*, 2008 ▶; Petoukhov *et al.*, 2006 ▶). The program *SASREF* operates with atomic models only and does not account for missing residues. In *BUNCH* (Petoukhov & Svergun, 2005 ▶), a multidomain protein may contain rigid domains with known structure connected by chains of DRs playing the role of missing flexible linkers. SA is employed to find the optimal arrangement of the domains and probable configuration of DR linkers fitting the SAXS data whereby the conformational space is explored by random rotation of model portions around randomly selected DRs.

The two approaches have a broad range of individual applications; however, a number of systems require a combination of these, for example, complexes consisting of multiple subunits where one or more components have missing fragments of noticeable length. In such a case neither *SASREF* nor *BUNCH* are applicable in their original form. *SASREF* could not be applied as it does not account for missing portions, and *BUNCH* can only handle one polypeptide chain (per asymmetric part). A new approach implemented in the program *CORAL* (complexes with random loops) has been developed to fill this gap (Fig. 3 ▶ *c*). *CORAL*, similarly to *SASREF*, translates and rotates the atomic models of individual domains belonging to multiple components of the complex. The difference is that these rearrangements are not fully random: the distances between the N- and C-terminal portions of the subsequent domains belonging to one chain are constrained. For this purpose a library of self-avoiding random loops composed of DRs is generated for the linker lengths from 5 to 100 amino acids using the *RANLOGS* tool, sampling 20 structures for every possible end-to-end distances for the given length with the binning step of 2 Å. When a domain is moved in *CORAL* its new position is examined by querying the library; if a linker of appropriate length to connect this domain with the preceding/following one cannot be found, then such a movement is rejected. If the query is successful the corresponding random loop is inserted as a placeholder of the missing linker and its contribution is added to the computed scattering intensity of the system and to the target function (*e.g.* overlaps, contact restraints *etc.*). C- and N-terminal portions of the subunits, if missing, can also be randomly selected from the library, but they do not constrain the associated domain motion. A feature of consorted motion of domains (missing in *SASREF* and *BUNCH*) is introduced so that the selected domains keep their mutual arrangement while changing their position and orientation with respect to the rest. This feature is useful, for example, when there are several known oligomerization interfaces in one system.

### 6.3. Characterization of conformational variability by SAXS   

SAXS is one of a few structural methods applicable to flexible biological systems including intrinsically disordered proteins and multidomain proteins with flexible linkers (Bernadó *et al.*, 2007 ▶; Bernadó, 2010 ▶; von Ossowski *et al.*, 2005 ▶). For these systems, interpretation of the scattering data in terms of a single model is not feasible because of significant conformational polydispersity. In the *ATSAS* package, quantitative analysis of such systems is performed using the ensemble optimization method (EOM; Bernadó *et al.*, 2007 ▶), which allows for a co-existence of multiple conformers in solution.

In EOM, the analysis of a potentially flexible system using scattering data is carried out in two steps: (1) A large pool of random configurations (genes) is first generated by the program *RANCH*, utilizing high-resolution models for regions of known structure when available and DRs for the flexible segments. (2) Ensembles of candidate conformations (chromosomes) are selected from this pool by a genetic algorithm, *GAJOE*, such that the average computed scattering over the ensemble fits the experimental scattering data (Bernadó *et al.*, 2007 ▶). If the 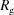 distribution of the models in the selected ensembles is as broad as that in the initial random pool, the protein is likely to be flexible; obtaining a narrow 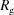 distribution peak suggests that the system is rigid (Bernadó, 2010 ▶). Furthermore, the position of the distribution of a selected ensemble relative to the pool provides an indication of the degree of compactness or the extended nature of the system.

In the new release, accounting for requests from the user community, the pool generation procedure in *RANCH* is generalized to include oligomeric multichain proteins (Fig. 3 ▶ *d*). For such objects, the multimerization interface and/or the structure of the contact domains is often known and EOM is applied to analyse the flexibility of the entire macromolecule while fixing the core belonging to the interface. The latest version of *RANCH* allows for the generation of random pools of oligomeric multichain conformations, with options for the incorporation of symmetry and the specification of inter-domain contacts. The rigid bodies describing the protein are defined for the initial chain generation, and the oligomerization interface is specified through a set of user-defined contacting residues and distances. A known interface in the case of symmetric multimers can also be maintained by fixing the monomer in the proper position and orientation yielding the correct arrangement of the other monomers when the symmetry operations are applied. *RANCH* generates a random conformation of the reference chain and applies the symmetry operation to construct the rest of the macromolecule. Models yielding steric clashes are rejected such that the pool contains only physically sound conformations. The original genetic algorithm implemented in *GAJOE* has also been optimized whereby an automatically adjustable size of the chromosome is used. This allows for an improved assessment of the conformational variability of the ensemble compared to the fixed number of genes in the chromosome.

## 7. Extension of online services and provision of indexed help tool   

### 7.1. *ATSAS* documentation   

**Figure 4:**
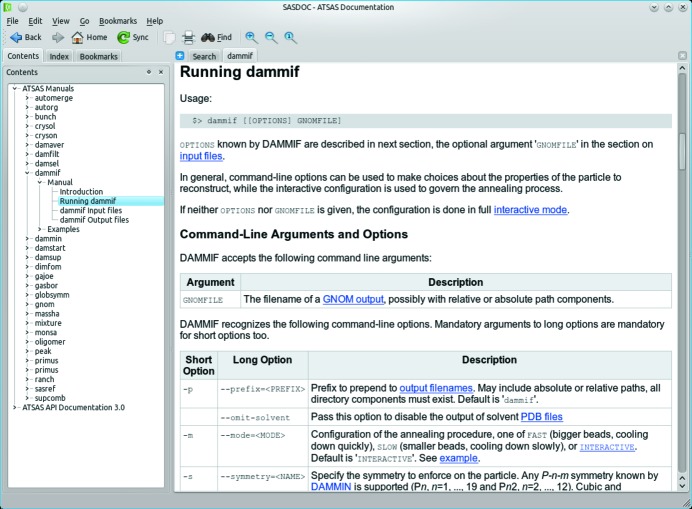
Screenshot of *SASDOC* showing the list of *ATSAS* manuals available. The highlighted entry in the contents pane (left) is shown in a tab in the main part of the window. A built-in full text search, an index and bookmarks are available.

With the further growth and increase of functionality of *ATSAS*, provision of a convenient user documentation system available on multiple platforms became a necessity. In the present release, a comprehensive documentation of the programs has been recompiled and a browser-like tool, *SASDOC*, has been developed to present the online documentation. *SASDOC* is based on the Qt Assistant (http://doc.qt.nokia.com/latest/assistant-manual.html) and provides full access to the *ATSAS* documentation, including built-in full text search, index and bookmarks. Fig. 4 ▶ shows a screenshot of *SASDOC* presenting the manual of *DAMMIF* (Franke & Svergun, 2009 ▶). Multiple tabs may show different parts of the manuals. Multiple Qt compiled help files may be bundled into a Qt help collection, which allows one to register additional help files with the *ATSAS* help collection to include locally available documentation into *SASDOC*.

### 7.2. Forum for *ATSAS* users   

A SAXIER forum (http://www.saxier.org/forum) was launched to facilitate exchange of information within the SAS community. The forum is maintained by the *ATSAS* developers and contains sections devoted to the *ATSAS* software package as well as SAS-related software and hardware issues. Since its start in August 2007, the forum has become the major web resource for information exchange about the use of SAS data analysis software (primarily *ATSAS*). In addition, the forum contains sections where SAS courses, workshops and new software releases are announced.

In August 2011, the forum had more than 570 registered participants from various institutes from all over the world. Of these participants, 25% were returning users who made three or more posts and thereby contributed 89% of the forum content; 33% of the participants registered to ask a specific question and made only one or two posts each. The forum has over 670 discussions composed of 2200 posts in total (the content is visible for unregistered guests as well). According to Google Analytics in the first half of 2011 the forum had about 2800 unique visitors per month, who made 11 000 page views; this is a 50% increase as compared to the same period of 2010. The main source of the traffic is Google (66%), which shows that the forum is well optimized for search engines. The forum runs on the free phpBB engine (http://www.phpbb.com) and uses the free anti-spam ACP module (http://www.lithiumstudios.org).

The forum provides an efficient way to support the *ATSAS* users and is becoming increasingly popular in the SAS community. At the same time the forum is an important source of feedback from the users to the *ATSAS* developers for further improvement of software and hardware in the future.

### 7.3. *ATSAS* online access   

The online interfaces to the major *ATSAS* programs are provided at the EMBL web site (http://www.embl-hamburg.de/biosaxs/atsas-online/), which enables remote job submission to the EMBL computational facilities. Originally (Petoukhov *et al.*, 2007 ▶), the interfaces to the *ab initio* programs *DAMMIN* (Svergun, 1999 ▶) and *GASBOR* (Svergun *et al.*, 2001 ▶) and atomic structure-based programs *CRYSOL* (Svergun *et al.*, 1995 ▶) and *SASREF* (Petoukhov & Svergun, 2005 ▶) were provided. The online access was recently opened also to the optimized shape determination algorithm *DAMMIF* (Franke & Svergun, 2009 ▶), the multiphase bead modelling program *MONSA* (Svergun, 1999 ▶; Svergun & Nierhaus, 2000 ▶) and the EOM method (Bernadó *et al.*, 2007 ▶). The *DAMMIF* interface enables multiple runs of the program followed by clusterization of the individual solutions by *DAMCLUST*. The two latter interfaces are especially convenient for less experienced users to enter the data and parameters for these tools, which require a rather cumbersome input in the standalone versions. Furthermore, the *CRYSOL* interface now allows one to submit multiple PDB files (in a ZIP archive) to be screened against the same scattering data set. Given the rather high speed of *CRYSOL* compared to the other *ATSAS* online tools, its jobs are now going *via* a fast track, being submitted to a separate queue on the cluster to avoid possible waiting in case of a high load of remote jobs. The applications available *via* the *ATSAS* online service are regularly updated in line with the entire package development and they are equivalent to the downloadable standalone versions in terms of functionality. The available program options defined by the choice from the web interface are reduced to the most typical cases in order to keep the online forms simple and convenient to use.

The *ATSAS* online service is rapidly increasing in popularity, with about 200 new users per year and a near-exponential growth of the number of jobs submitted. In the period from August 2007 to the end of 2011, over 800 scientists from about 400 laboratories used *ATSAS* online and submitted over 40 000 remote jobs.

### 7.4. *ATSAS* grid facilities   

In view of the rapidly growing number of users and jobs submitted to the *ATSAS* online service, the currently employed EMBL BioSAXS cluster (120 Opteron CPUs) does not have the capacity to keep up with user demand. Thus, in the framework of the WeNMR project (http://www.wenmr.eu), the *ATSAS* online service is being transformed to grid portals to make use of the grid, a large-scale international aggregation of computation and storage resources (http://www.embl-hamburg.de/biosaxs/atsas-grid). In the progress of the eNMR/WeNMR projects, a number of such portals were already set up for important NMR applications. One of the future aims of WeNMR is to unify access to all portals of the project and include access to major computational SAXS resources. The present *ATSAS* software package has already been installed on all compute elements that the enmr.eu virtual organization has access to. Users with their own grid certificates registered at http://www.enmr.eu/ can also directly access the various programs of the *ATSAS* suite and use them for their own computations, independent of the portals.

## 8. Conclusions   

In the present paper, only major recent enhancements in *ATSAS* were described. Most of the *ATSAS* programs are being constantly developed, and we are leaving aside some minor improvements. The *ATSAS* developments described in this communication focus not only on the design of the new algorithms but also on improvements in speed, convenience of use and, wherever possible, automation of the existing programs. The latter is a necessary prerequisite for meeting the challenge of the new brilliant synchrotron and spallation neutron sources able to provide immense amounts of experimental data within a short time frame. We plan to further automate and link together the *ATSAS* modules to provide a comprehensive SAXS/SANS data analysis system able to generate and rank structural models with minimal user intervention.

## Supplementary Material

. DOI: 10.1107/S0021889812007662/fs5015sup1.pdf
Supplementary table and figures

## Footnotes

1 Supplementary material for this paper is available from the IUCr electronic archives (Reference: FS5015). Services for accessing this material are described at the back of the journal.
