# Supplementary material for: New developments in the ATSAS program package for small-angle scattering data analysis
Source: J Appl Crystallogr. 2012 Mar 15;45(Pt 2):342–50. doi: 10.1107/S0021889812007662 (PMC4233345; doi:10.1107/S0021889812007662)
Supplement: Supplementary file 1 [file j-45-00342-sup1.pdf]

Method	Vdammin/MM			Vdammif/MM			Va/MM		Vp/MM
	# Shannon Channels			# Shannon Channels			DAMMIN	DAMMIF	
	8	10	12	8	10	12			
**X-ray**	1.73±0.27	1.67±0.22	1.64±0.23	1.76±0.21	1.61±0.28	1.56±0.33	1.70±0.19	1.66±0.22	1.65±0.23
**CEM**	1.75±0.46	1.75±0.36	1.65±0.37	1.80±0.43	1.65±0.41	1.59±0.41	1.72±0.39	1.68±0.40	1.62±0.42
**NMR**	1.67±0.24	1.65±0.27	1.56±0.23	1.70±0.21	1.55±0.29	1.56±0.30	1.62±0.23	1.61±0.23	1.55±0.23
**All**	1.73±0.31	1.68±0.26	1.63±0.26	1.76±0.26	1.61±0.30	1.56±0.34	1.69±0.25	1.65±0.26	1.62±0.28

Table S1. Excluded volume calculation by DAMMIN and DAMMIF for simulated data. *Va* is an average volume calculated by DAMMIN or DAMMIF for different number of Shannon channels. *Vp* is a Porod volume estimated by AUTOPOROD.

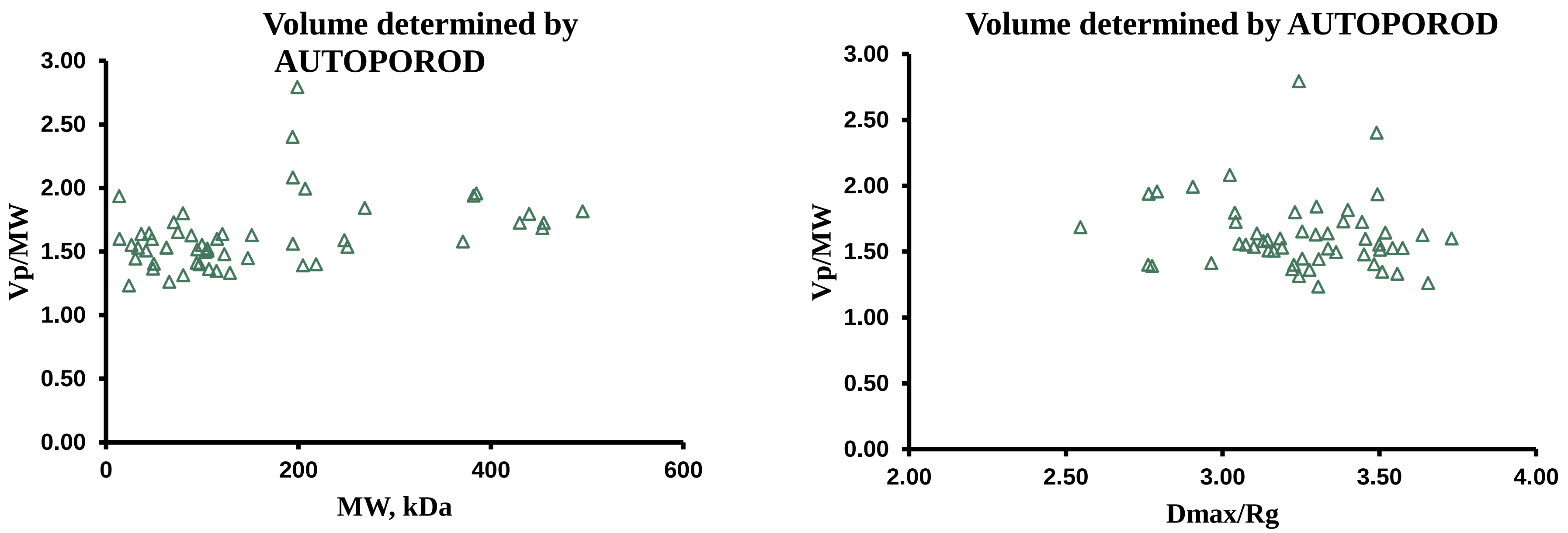

Figure S1. Correlation between molecular weight (MW) and Porod volume calculated by AUTOPOROD. On the left figure, volume calculated by AUTOPOROD divided by MW is plotted versus MW. On the right figure, Porod volume divided by MW is plotted versus maximum particle dimension (*Dmax*) divided by *Rg*.

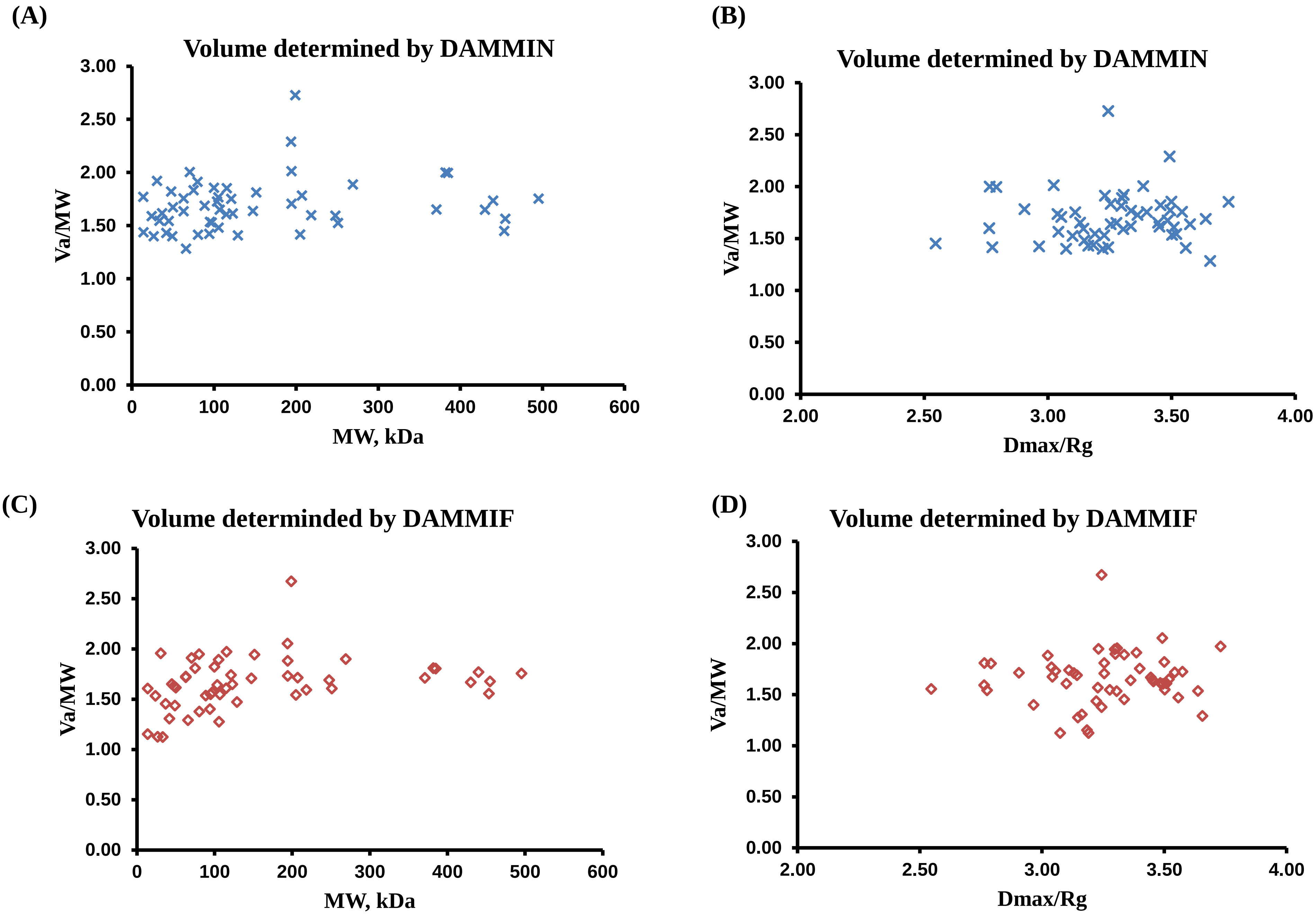

Figure S2. Correlation between molecular weight (MW) and excluded volume calculated by DAMMIN (top figures: A and B) and DAMMIF (bottom figures: C and D). On the left, average volume divided by MW is plotted versus MW. On the right, average volume divided by MW is plotted against maximum particle dimension (*Dmax*) divided by radius of gyration (*Rg*).
